# Predictive Value of Pretreatment BCR-ABL^IS^ Transcript level on Response to Imatinib Therapy in Egyptian Patients with Chronic Phase Chronic Myeloid Leukemia (CPCML)

**Published:** 2013-03

**Authors:** Wafaa H. El-Metnawy, Mervat M. Mattar, Yasser H. El-Nahass, Mohamed A. Samra, Hala M. Abdelhamid, Raafat M. AbdlFattah, Ahmad R. Hamed

**Affiliations:** 1 *Clinical Oncology Center, School of Medicine, Cairo University, Egypt;*; 2 *Department of Hematology, School of Medicine, Cairo University, Egypt;*; 3 *Department of Clinical Pathology, National Cancer Institute, Cairo University, Egypt;*; 4 *Department of Medical Oncology, National Cancer Institute, Cairo University, Egypt;*; 5 *Pharmaceutical Research Group, National Research Center, Cairo, Egypt*

**Keywords:** CPCML, BCRABL transcript, cytogenetic, molecular responses

## Abstract

**Background:**

A wide range of responses of patients with CPCML to IM has been reported. Several factors were proposed to predict response including molecular response at 3 and 6 months. Purpose: To study the impact of pretreatment BCR-ABL transcript level on molecular response to IM, and to assess the value of the milestone ; ≤10% transcript at 3 months on PFS and OS.

**Patients and Methods:**

Fifty five adult CP-CML patients receiving daily dose of 400 mg IM were subjected to molecular and cytogenetic analysis at diagnosis and at regular time intervals. Median follow up period was 36 months (15-48). Hematologic, cytogenetic, and molecular responses were rated according to ELN.

**Results:**

Two Patient groups were distinguished regarding response to IM therapy. A group of 22/55 patients (40%) having pretreatment BCR-ABL^IS^ level ≤200% and a second patient group 33/55 (60%) having transcript level >200%. The ≤10% milestone was achieved by 15/22 patients (68%) versus 7/33 patients (21%), *p*=0.04 in favor of the first group. Optimal responders in first group were 14/22 (64%) compared to 13/33 (39%) in second group, *p*=0.02. Achievement of 10% transcript level significantly correlated with longer PFS. The median BCR-ABL^IS^ transcripts levels in optimal responders at 3, 6 and 18 months was 10%, 2% and 0.1%, respectively compared to 100%, 65% and 10%, in suboptimal/resistant patients *p*=0.001. Resistance in 11 patients was correlated with identifiable ABL Kinase mutations.

**Conclusions:**

The Pretreatment 200% cutoff and the 3 month BCR-ABL^IS^ ≤10% transcript levels proved strong predictors of response to IM and significantly correlated with probability of CCyR, MMR and PFS.

## INTRODUCTION

The introduction of Imatinib Mesylate (IM) in 1998 has revolutionized the management of Chronic Myeloid Leukemia (CML). This selective tyrosine kinase inhibitor offers a durable response in a high percentage of patients with favorable long term safety profile and decreasing rate of relapse (1). Impressive response rates and good tolerability have made IM the standard front line therapy for CML. Despite its remarkable efficacy for the treatment of chronic phase (CP) CML, a growing number of patients either fail IM therapy due to emergence of ABL kinase domain (AKD) mutations or develop intolerance to the drug (2). All treatment trials with IM insisted on the close sequential molecular monitoring of patients under IM therapy to assess the kinetics of BCR ABL transcripts level regression and its impact on the remote cytogenetic response and progression free survival (PFS) (3-5). We have previously reported that patients achieving two log reduction of BCR-ABL transcript level after 6 months of IM therapy could benefit of a sustained cytogenetic and molecular responses (6). Several reports have focused on the correlation between earlier molecular response at 3 months and the probability of longer disease free survival (DFS) and higher rate of overall survival (OS) (7, 8). A BCR-ABL^IS^ transcript level of ≤10% after 3 months or <1% after 6 months of IM therapy has been found to be associated with significant increase of the probability of obtaining a complete cytogenetic (CCyR) and molecular (CMR) responses (9). In addition, a high BCR-ABL^IS^ transcript level at diagnosis was found to be related to a non-optimal response to IM therapy (7).

In the present study we aimed at

1. Exploring the relation between pretreatment BCR-ABL^IS^ transcript level and molecular and cytogenetic responses to IM.
2. Assessing the impact of achieving ≤10% BCR-ABL^IS^ transcripts level at 3 months of IM therapy on progression free survival (PFS) and overall survival (OS) in a cohort of Egyptian patients.

## PATIENTS AND METHODS

### Study group

Fifty five adult Egyptian patients with CP-CML were evaluable for molecular analysis at diagnosis and at 3 months interval of IM therapy. The patients presented to the outpatient clinics of the Medical Oncology Unit, National Cancer Institute (NCI) and hematology Unit of the Medical school, Cairo University from May 2007 to May 2011. The study was carried out according to declaration of Helsinki and approved by the institutions review boards. Informed consent was provided by all patients.

All patients had morphologic and cytogenetic evidence of Philadelphia positive CP-CML (defined as less than 12 months from diagnosis); age 18 years or older; having normal blood chemistry and normal cardiac function. Women at childbearing age were required to have a negative pregnancy test before starting IM and to use contraception during therapy. Excluded from the study were patients receiving CML therapies other than IM (busulfan, IFN-α, or Ara-C). Exceptions included anagrelide and hydroxyurea for the treatment of elevated platelet (>700 × 10^9^/L) and WBC count (>50 × 10^9^/L), respectively; for more than 4 weeks.

### Treatment

Patients were treated within an international Novartis-sponsored protocol with Imatinib Mesylate (IM) at a daily oral dose of 400 mg. The dose was reduced to 300 mg for any ≥ Grade 3 drug-related hematologic toxicity. No dose adjustments were made for grade 1 or 2 hematologic toxicities.

### Methods

BCR-ABL^IS^ transcript level according to International Scale (IS) was determined usingReal Time quantitative polymerase chain reaction (RQ-PCR) detection system (*Ecco Illumina, USA*). The *ABL* control gene and the *BCR-ABL* target gene transcript levels were measured using Universal PCR Master Mix (*Nanogen advanced diagnostics, SrL, Italy*) and specific primers and hydrolysis probe (*Philadelphia p210 and Abl QPCR alert Amplimix and QPCR alert Ampliprobe*).A set of reference RNA was usedto convert results into IS. Quantitations were made against specific Abl and p210 standards (*Philadelphia p210 QPCR standards, Nanogen*) (10). Cytogenetic analysis was performed by G banding technique according to standard methods (11). At least twenty metaphases were analyzed for each sample. Fluorescent insitu hybridization (FISH) analysis for BCR and ABL genes using probes from *Vysis Inc.* (*Downers Groove, IL, USA*). A minimum of 100 interphase nuclei were evaluated for each patient.

### Mutation Screening

Genomic DNA was extracted from peripheral blood using Gentra Puregene blood kit (*QIAGEN, Hilden, Germany*). ASO-PCR assay was established for the detection of 16 known mutations which were selected according to their frequency in IM-resistant CML patients. Mutation panel selected in this study included M244V, L248V, G250E, Q252H (a), Q252H (b), Y253H, Y253F, E255K, E255V, V299L (a), V299L (b), F311L, T315I, F317L, M343T, M351T, E355G and F359V. Mutated or wild-type sequences were specifically amplified in a PCR reaction to analyze the most frequently identified mutations in the AKD (amino acids 220 to 498). The amplified products were detected by electrophoresis on 2% ethidium bromide-stained agarose gel (13, 14).

### Definition of treatment responses

Hematologic, molecular and cytogenetic responses were defined according to European Leukemia Net criteria. MMR was defined as a 3-log reduction of BCR-ABL transcripts level, corresponding to ≤0.1% on IS (12). Progression free survival (PFS) defined as loss of hematologic or cytogenetic response, death, or development of advanced CML. Overall (OS) defined by the absence of death from any reason (15).

### Statistical analysis

All analyses were performed using the statistical package for the social sciences (SPSS software 17; SPSS Inc., Chicago, USA). We used a receiver operating characteristic curve (ROC) to identify the cutoffs in pretreatment transcript level that would best predict patient outcome (16).

## RESULTS

Fifty five Egyptian patients; 33 males and 22 females were enrolled in this study. Median age of the whole cohort at diagnosis was 40.5 years (19-60). Median total leukocytic count was 150 × 10^9^/L (27-583). Median hemoglobin and platelet count was 11.4 gm/dl (8.2-16.9) and 340 × 10^9^/L (115-1000), respectively. Median follow up period was 36 months (15-48). Complete hematologic response (CHR) at 3 months of IM therapy was achieved by 54/55 patients (98.2%). Complete Cytogenetic Response at 12 months and MMR at 18 months of IM therapy were achieved by 28/55 patients (51%).

### Pretreatment BCR-ABL^IS^ transcript level and response to IM

According to pretreatment BCR-ABL^IS^ transcript level, two groups of patients could be distinguished regarding response to IM therapy. One group of 22 patients (40%) having a pretreatment BCR-ABL^IS^ ≤200% (26-200%, median 75%) and a second group of 33 (60%) patients with a pretreatment BCR-ABL^IS^ transcript level >200% (223-2000%, median 470%) (Table 1). Subgroup analysis of both groups revealed that at 3 months of IM therapy, 15/22 patients (68%) of the first group achieved a BCR-ABL^IS^ transcript level of ≤10% versus 7/33 patients (21%) in the second group, *p*=0.04. At 3 months of IM therapy, transcript level of 1-5% was achieved by 7/22 patients (32%) of first group versus 1/33 patients (3%) of the second group (*p*=0.01).

**Table 1:** Effect of Pretreatment BCR-ABL transcript level on Molecular Response to IM

Pretreatment BCR-ABL^IS^ Transcript level	≤200%	>200%	*p-*value

No. of patients	22	33	
Median % BCR-ABL^IS^ transcript level (Range)	75 (26-200%)	470 (223-2000%)	0.007
No. of Patients with ≤10% BCR-ABL^IS^ Transcript at 3 Mo (%)	15 (68%)	7 (21%)	0.04
No. of Patients with 1-5% BCR-ABL^IS^ Transcript at 3 Mo (%)	7 (32%)	1 (3%)	0.01
No. of Patients with optimal response; [CCyR at 12 months, MMR at 18 months] (%)	14 (64%)	13 (39%)	0.05

The number of optimal responders in patients with pretreatment BCR-ABL^IS^ ≤200% was 14/22 (64%) compared to 13/33 (39%) in the patient group with >200% (*p*=0.05) (Table 1).

The median BCR-ABL^IS^ transcripts level in optimal responders at 3, 6 and 18 months was 10%, 2% and 0.1%, respectively. On the contrary, patients exhibiting refractoriness and suboptimal response showed a significantly sluggish regression of the median transcript levels, at the corresponding time points (100%, 65% and 10%), (*p*=0.001) (Table 2).

**Table 2:** Correlation between BCR ABL Transcript Regression and Response to IM

Response Group	Median BCR-ABLIS % at diagnosis (range)	Median BCR-ABLIS % at 3 months (range)	Median BCR-ABLIS % at 6 months (range)	Median BCR-ABLIS % at 18 months (range)

Optimal (n:28)	240.0 (26.0-820)	10.0 (1.0-100)	2.0 (0.1-11)	0.1 (0.003-0.3)
Sub-optimal & resistant (n:27)	380.0 (34.0-2000)	100.0 (4.0-580)	65.0 (2.0-3000)	10.0 (1-287)
*p*-value	0.09	<0.001	<0.001	<0.001

### BCR-ABL^IS^ 10% milestone at 3 months and PFS /OS

**Figure 1:**
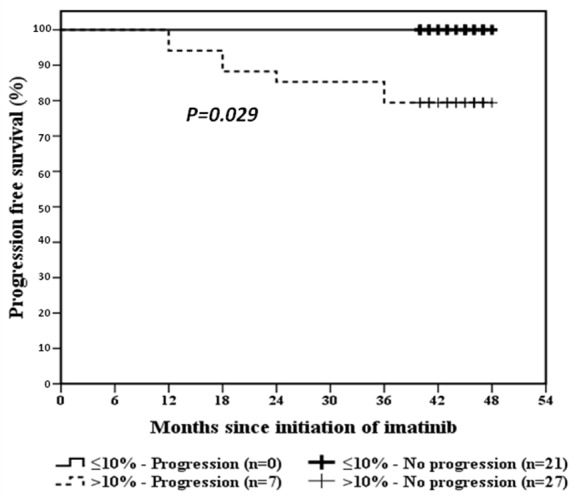
PFS curves of BCR-ABL^IS^ ≤10% and >10% at 3 months of IM therapy

**Figure 2:**
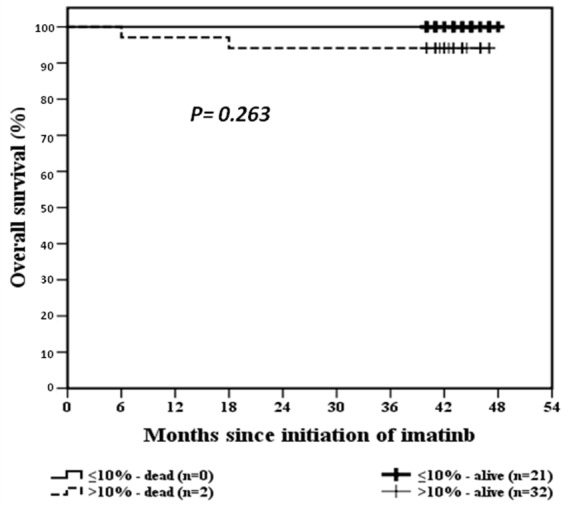
OS curves for BCR-ABL^IS^ ≤10% and >10% at 3 months of IM therapy

The rates of PFS and OS of the whole study group at 3 years of IM therapy was 87% and 96.4%, respectively. The estimated PFS at 3 years for patients who achieved the ≤10% at 3 months of IM therapy was 100% versus 80% for patients with >10% (*p*=0.02), Figure 1. OS for patients according to the 10% BCR-ABL^IS^ transcript level at 3 months of IM therapy is demonstrated in Figure 2.

### Mutational status of resistant patients to IM

Eleven patients (11/55, 20%) have shown resistance to IM and experienced disease progression to acute blastic crisis (ABC) in 3 patients, to accelerated phase (AP) in 2 patients and 2 disease related deaths. None of these patients had demonstrated decrease in transcript level to 10% at 3 months of IM treatment. Mutational analysis revealed P-loop mutations (amino acids 248-255) in 6/11 of patients including Q252Ha and Q252Hb in 3 patients, Y253H in 1 patient and 2 patients having E255K. Non-P loop mutations were found in 4 patients, 3 patients with M351T, one patient with F359V and one patient without any of studied mutations. None of these patients was harboring the gatekeeper mutation T315I.

## DISCUSSION

Although CCyR has long been the gold standard of response and survival to IM treated patients with CPCML (17) yet, we and others have stressed the importance of the kinetics of molecular response to IM (3, 6). The application of an international standardization of BCR-ABL^IS^ transcript level enabled a consistent monitoring of patients and more appropriate assessment of the deep molecular responses during the early phase of treatment (4). Most studies have demonstrated that response determined by BCR-ABL^IS^ transcripts level at defined time points has been the more reliable predictive parameter of long term evolution of the disease (7, 8). We have previously reported the predictive value of 2 log reduction of BCR-ABL transcript level after 6 months of IM therapy on PFS and sustained cytogenetic response (6). Recently the kinetics of molecular response at 3 months became a more rising predictive issue (7, 8, 18) where the 3-month BCR-ABL^IS^ ≤10% transcript level was evoked as a strongest predictor milestone of response (9).

In the present study of an Egyptian cohort of 55 CPCML patients (40%) achieved BCR-ABL^IS^ ≤10% transcript level after 3 months of IM therapy. Subgroup analysis of these 22 patients revealed that 15/22 patients (68%) had a pretreatment BCR-ABL^IS^ transcript level ≤200% and 7/33 patients (21%) had a pretreatment level >200% suggesting that the cutoff of 200% could also have a significant impact on the kinetics of molecular response. These data concur with previously reported (7). Complete cytogenetic response and MMR at their specified times were significantly associated with achievement of the 3 months milestone BCR-ABL^IS^ ≤10%, *p*<0.001. According to some reports, about one-fourth of CML patients were declared high-risk at 3 months (with BCR-ABL^IS^ >10%) and the same proportion of treatment failure was also observed in the IRIS study. This led to a conclusion that failure of achieving ≤10% BCR-ABL^IS^ transcript level after 3 months of IM therapy will lead to disease progression and treatment failure (8). According to failure of achieving this 3 months BCR-ABL^IS^ ≤10% milestone 33/55 patients (60%) in the present study would be considered high risk. However it is importantly to say that among them 7 patients (13%) were optimal responders at 18 months which may raise the issue of distinct biological characteristics of slow responders, such as low hOCT1 or relatively high MDR1 expression (19-21). Both optimal responders and slow responders behaved equally regarding PFS and OS, which is in concordance with our previously reported data on the predictive value of 2 log reduction at 6 months of IM therapy on PFS and OS in another Egyptian series (6). This also stresses on the importance of postponing shift to second generation tyrosine kinase inhibitors (G2TKIs) as changing treatment to second line TKIs after only 3 months would be unusual. Whether an inferior response at 3 months will trigger treatment revision on a considerable proportion of patients should be considered on basis of other biological factors that would affect response.

The data in the present study showed that resistance and disease progression occurred in 11/55 (20%) patients who had both higher pretreatment transcript level and in the same time failed to achieve the BCR-ABL^IS^ ≤10% milestone after 3 months of IM . These data point to the pretreatment transcript level as an additional predictive factor of the type of molecular response to IM. However and more importantly the concomitant occurrence of both factors might trigger performing mutational analysis of AKD which may prompt a change of therapeutic strategy , especially if the encountered mutations are Known to be sensitive to second generation Tyrosine Kinase inhibitors (G2TKI) as in the present study.

Although a lower fraction of our patients could achieve the 3 months BCR-ABL^IS^ ≤10% transcript than reported by others (40% versus 64%) (7, 8) yet, optimal response rates at 18 months were not fairly different (51% versus 60%). Our patients seem to be slow responders as shown by median BCR-ABL^IS^ transcripts level in optimal responders at 3, 6 and 18 months (10%, 2% and 0.1%, respectively) compared to others (7, 8). No significant difference in OS at 3 years between patients with 3 month BCR-ABL^IS^ ≤10% and >10% (100% versus 96%, *p=0.2*). However, we and others, demonstrated a lower rate of 3 year PFS in the >10% group (100% and 80%,* p=0.02* (8).

In conclusion, our data point to the pretreatment 200% cutoff transcript level as an additional predictive factor of the type of molecular response to IM and add further proof to the 3 month BCR-ABL^IS^ ≤10% transcript level as a strong predictor of response and correlates significantly with probability of achieving CCyR and MMR at their specified time points with higher estimates of PFS.
